# The association between early-life respiratory infections and childhood asthma: a meta-analytic perspective

**DOI:** 10.3389/fmed.2026.1751843

**Published:** 2026-02-16

**Authors:** Rong Zhao

**Affiliations:** Traditional Chinese Medicine Pediatrics, The Central Hospital of Enshi Tujia and Miao Autonomous Prefecture, Enshi, Hubei, China

**Keywords:** childhood asthma, early-life respiratory infections, maternal vaccination, RSV prophylaxis, wheezing

## Abstract

**Background:**

Early-life exposures and interventions establish essential pathways which determine children’s development across respiratory, allergic, and growth pathways. The scientists conducted a systematic review and meta-analysis which combined multiple studies to examine how nutritional interventions and viral prophylaxis and probiotics and environmental exposures and allergen exposures and clinical factors in infants affect asthma and wheeze and atopy and growth outcomes.

**Methods:**

The research team examined 51 studies which they categorized into eight different research areas. The researchers conducted random effects meta-analyses with inverse variance method to calculate pooled odds ratios (ORs) which included 95% confidence intervals (CIs). The researchers used I^2^ statistics to measure heterogeneity while they used funnel plots and Egger’s test to assess publication bias.

**Results:**

The nutritional interventions during pregnancy (OR = 0.89, 95% CI: 0.74–1.07, *I*^2^ = 68%) and early probiotics/microbiota modulation (OR = 1.10, 95% CI: 0.84–1.43, *I*^2^ = 96%) failed to show any significant results. The combination of viral prophylaxis with maternal influenza vaccination protected against wheeze and asthma and LRTI development (OR = 0.78, 95% CI: 0.72–0.85, *I*^2^ = 68%). The presence of early-life respiratory viral infections raised the likelihood of developing wheeze and asthma (OR = 1.59, 95% CI: 1.39–1.82, *I*^2^ = 90%) which accompanied allergen/atopy exposures (OR = 1.40, 95% CI: 1.30–1.50) and environmental exposures (OR = 1.34, 95% CI: 1.27–1.42) and infant clinical factors (OR = 1.29, 95% CI: 1.13–1.46, *I*^2^ = 82%). The long-term cohort studies established that early-life risk factors maintain a consistent impact on the development of asthma and allergic conditions (OR = 1.41, 95% CI: 1.35–1.48). The studies on viral prophylaxis and respiratory infections and allergen exposure demonstrated evidence of publication bias.

**Conclusion:**

Research shows that early-life exposure to viral infections and allergens and environmental pollutants and infant clinical factors establishes strong links to the development of respiratory disorders and allergic diseases in children. The nutritional and probiotic treatments produced restricted and unstable results which demonstrate the requirement for specific preventive methods that should be implemented during early life.

## 1 Introduction

The early factors of respiratory susceptibility are getting more attention as childhood asthma remains a significant worldwide health concern (1–5). A large amount of epidemiological data suggests that the path toward asthma may begin in infancy, a time of development when the immunological and respiratory systems are more vulnerable to outside disturbances. Acute respiratory infections, especially those brought on by RSV, influenza, and other viral pathogens, have become prominent early-life exposures that can influence prolonged airway outcomes among these disruptions (6–9). Prophylactic trials have demonstrated that averting serious RSV illness in infancy lowers recurrent wheezing and hospitalization, making early-life RSV encounter one of the most frequently implicated viral determinants (10–12). In a similar vein, it has been demonstrated that maternal immunization can modify influenza-related respiratory morbidity in infancy, resulting in a considerable reduction in hospitalizations for respiratory tract infections and newborn influenza cases (13–15). The concept that early respiratory infections have significant downstream impacts on airway health and the occurrence of asthma is backed by all of these data.

Trials assessing perinatal nutritional modification offer further mechanistic knowledge. The idea that prenatal immunological programming affects postnatal respiratory consequences is supported by research showing that fish oil intake during pregnancy reduces chronic wheezing and asthma in children (16–19). Similar directionally positive impacts on early wheezing are shown by vitamin D trials, indicating that biological processes associated with infection susceptibility also intersect with asthma incidence (20–23). The interdependence of microbial exposures, immunological maturation, and atopic illness, all important components in asthma pathophysiology, is further highlighted by parallel data from early-life probiotic studies that show decreases in IgE-linked eczema and early atopic conditions (16, 17, 24–28).

Furthermore, studies focusing on airway physiology support the idea that early disruptions could affect subsequent respiratory traits. While high-intensity physical activity increases airway responsiveness in sedentary children (29–32), airway-hyperresponsiveness-guided asthma therapy enhances lung function metrics in school-age children (13–15, 18, 19). The basic foundation that unites these trials is that if early viral infection plays a role in the pathophysiology of asthma, then altering viral contact, infection intensity, or the host inflammatory milieu during initial stages might impact subsequent respiratory danger. Although individual trials provide valuable insights, overall interpretation is challenging due to variations in the nature, timing, magnitude, and outcome definitions. We compiled high-quality RCTs to investigate the potential link between early-life respiratory infections and subsequent wheezing and asthma in order to shed light on these trends. A variety of preventive measures targeted at altering early respiratory exposures are examined in this meta-analysis, which evaluates their cumulative connection with ongoing asthma-related consequences.

## 2 Methodology

### 2.1 Study design

This systematic review and meta-analysis were carefully designed and reported in accordance with the Preferred Reporting Items for Systematic Reviews and Meta-Analyses (PRISMA 2020) standards.

### 2.2 Search strategy

A thorough search was carried out using PubMed/MEDLINE, Scopus, Web of Science, Cochrane CENTRAL, and ClinicalTrials.gov from inception to December 2024. Search queries included controlled vocabulary (MeSH) and free-text terms such as: “early-life infection,” “respiratory tract infection,” “childhood asthma,” “wheezing,” “RSV prophylaxis,” “maternal vaccination,” “maternal supplementation,” “vitamin D,” “fish oil,” “probiotics,” “palivizumab,” “exercise intervention,.” Geographical or linguistic limitations were not imposed.

### 2.3 Eligibility criteria

Randomized controlled trials and observational studies that enrolled participants from prenatal to age 12 and assessed therapies that could change the timing, intensity, or tendency of early-life respiratory illnesses were eligible. Asthma, recurrent wheezing, respiratory hospitalization, atopic disease, or lung-function outcomes had to be reported in the trials, and they had to include extractable numerical data that could be used to calculate effect sizes using standardized mean differences. Studies that did not include early-life subjects, had limited outcome reporting, employed observational or quasi-experimental designs, or lacked a comparison group were disqualified.

### 2.4 Data extraction

A uniform template was used by a reviewer to extract data individually. Study design, sample size, intervention and control characteristics, geographic region, age at intervention or follow-up, respiratory infection type, severity characterization, definitions of asthma or wheeze outcomes, length of follow-up, and year of publication were among the variables that were obtained. To guarantee uniformity in stratified analyses, all retrieved variables were predefined. Conflicts were settled by dialog and agreement.

### 2.5 Quality assessment

The Cochrane RoB 2.0 tool was used to evaluate bias risk. The GRADE paradigm was used for rating the degree of certainty of the evidence, taking into account publication bias, indirectness, imprecision, risk of bias, and inconsistency.

### 2.6 Statistical analysis

A random-effects model with inverse-variance weighting was used to produce effect estimates. To account for discrepancies in outcome assessment between trials, OR and associated 95% confidence intervals were computed. To evaluate heterogeneity, the I2 statistic was employed. Age during infection or exposure, kind of respiratory illness, extent of infection, definition of asthma outcome, regional or socioeconomic context, length of follow-up, and publication time were all assessed in prespecified subgroup analyses. Publication bias was assessed via visual analysis of funnel plots. RevMan 5.4 was used for statistical analysis.

## 3 Results

### 3.1 Study selection

**Figure 1:**
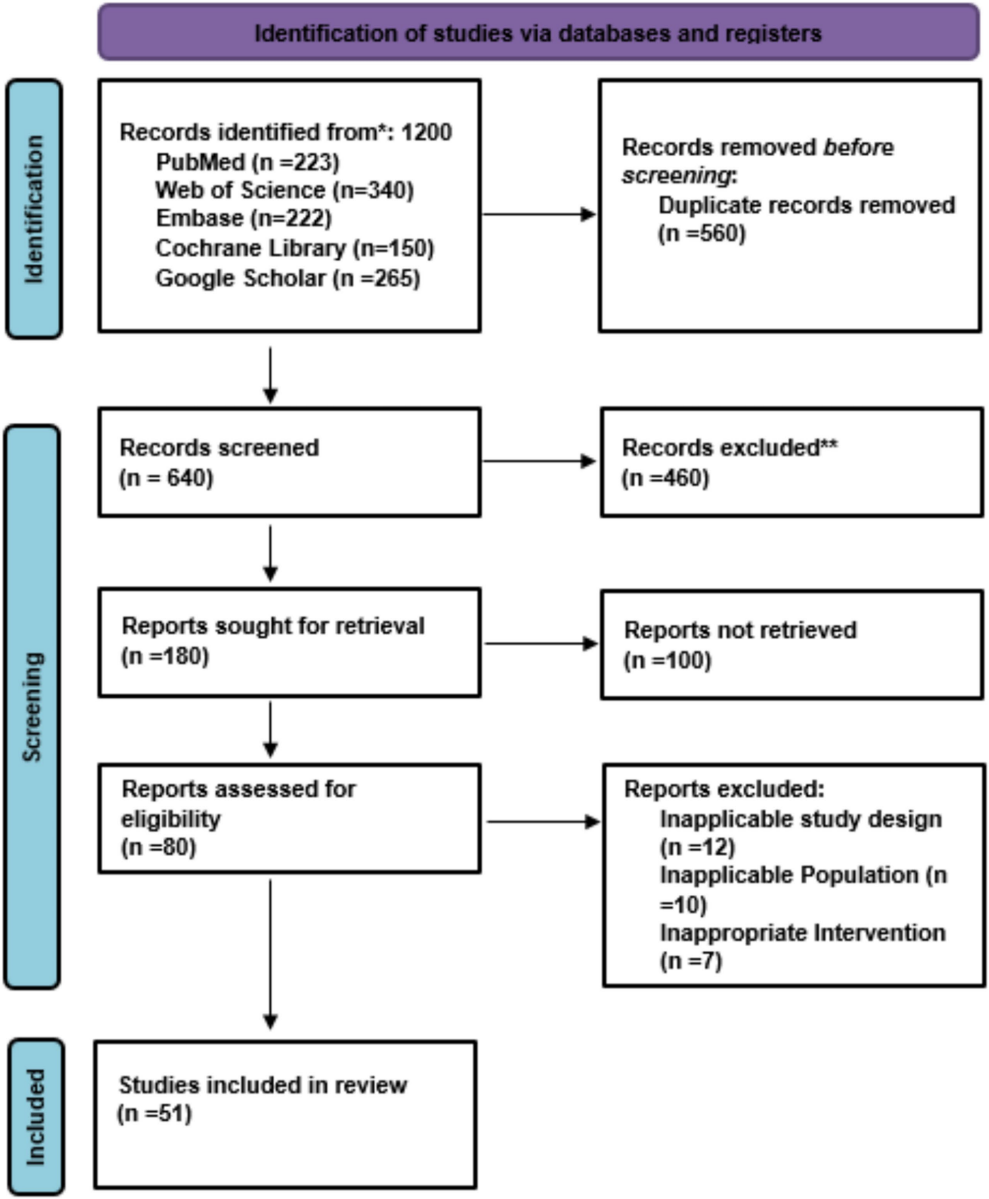
PRISMA flow chart of study selection.

The overall systematic search process from one of the databases identified a total of several records, out of which 51 randomized controlled trials and clinical cohort studies were found to meet the appropriate criteria for inclusion and hence were the ones considered for this meta-analysis. One the other hand, studies reporting no relevant outcomes (asthma, wheezing, or atopic disease), non-randomized designs, or no suitable comparators were excluded from the analysis. Among others, the exclusion of certain studies comprised early observational studies on RSV without outcome reporting, small pilot trials lacking statistical power and studies outside the relevant age range (<1 month or >12 years). Figure 1 illustrates a flow diagram of the search, screening, eligibility, and inclusion process (Figure 1).

### 3.2 Characteristics of studies on early-life respiratory infections and childhood asthma

The studies that examine how respiratory infections during early life affect the development of asthma in children use both experimental research methods and observational research methods. The randomized controlled trials (RCTs) tested various interventions on pregnant women and preterm infants and high-risk children who received fish oil-derived n-3 fatty acids and vitamin D, probiotics, and palivizumab, motavizumab, and influenza vaccination. The study enrolled participants with sample sizes that ranged between 132 to 2,116 who received interventions starting from mid-pregnancy until their first year of life or during their seasonal RSV/influenza exposure. The study assessed four different outcomes, including persistent wheeze and asthma incidence and recurrent wheezing and allergic diseases. The observational studies, which included prospective and longitudinal and registry-based and population-based cohort studies, followed children from their birth until they reached adulthood, with study participants numbering between hundreds and more than 1 million. The research studies investigated how natural environments affect people through their respiratory infections and allergen exposure, antibiotic consumption, air pollution, and home environmental conditions. The evidence from both experimental studies and observational studies shows that early-life infections and environmental exposures result in increased asthma risk and allergic conditions that persist throughout childhood and later life (Table 1).

**Table 1:** Baseline characteristics of the included studies.

Author(s)	Year	Country	Study Type & Population	Sample Size	Duration	Outcome
Bisgaard et al. (79)	2016	Denmark	Randomized, double-blind, placebo-controlled trial; pregnant women	736	From week 24 of pregnancy until 1 week after birth	Reduced risk of persistent wheeze or asthma in offspring by age 3 years
Litonjua et al. (41)	2014	USA	Randomized, double-blind, placebo-controlled trial; pregnant women	876	From 10–18 weeks gestation until delivery	Designed to assess primary prevention of asthma and allergies in children; results pending at study design stage
Roth et al. (35)	2018	Bangladesh	Randomized, double-blind, placebo-controlled trial; pregnant and lactating women	1,300	From 17–24 weeks gestation through 26 weeks postpartum	No significant effect on infant growth at 1 year; vitamin D levels increased safely
Simoes et al. (42)	2007	Multicenter (Europe & USA)	Randomized, placebo-controlled trial; infants born preterm	1,502	Monthly injections during RSV season	Reduced RSV hospitalization and subsequent recurrent wheezing in high-risk infants
Blanken et al. (36)	2013	Netherlands	Randomized, double-blind, placebo-controlled trial; healthy preterm infants	429	During first RSV season	Significantly reduced recurrent wheeze through first year of life
Feltes et al. (43)	2011	Multicenter (USA & Canada)	Randomized, double-blind, active-controlled trial; children with congenital heart disease	1,287	RSV season (monthly injections)	Motavizumab non-inferior to Palivizumab for preventing serious RSV disease
Madhi et al. (37)	2014	South Africa	Randomized, placebo-controlled trial; pregnant women	2,116	From 20–36 weeks gestation until delivery	Reduced influenza illness in mothers and infants during first 6 months
Mattila et al. (44)	2022	Finland	Randomized clinical trial; children <18 years with respiratory infection	1,000	During acute illness visit	Reduced unnecessary antibiotic use in children
Nunes et al. (38)	2017	South Africa	Randomized, placebo-controlled trial; pregnant women	2,116	From 20–36 weeks gestation until delivery	Reduced all-cause lower respiratory tract infection hospitalizations in infants
Scheltema et al. (39)	2018	Netherlands	Randomized, double-blind, placebo-controlled trial; healthy preterm infants	429	During first RSV season	Reduced RSV infection; no clear long-term effect on asthma development
Cabana et al. (45)	2017	USA	Randomized, double-blind, placebo-controlled trial; infants	184	First 6 months of life	Reduced eczema risk; trend toward lower asthma incidence
Abrahamsson et al. (46)	2007	Sweden	Double-blind, randomized, placebo-controlled trial; infants	230	First 6 months of life	Reduced IgE-associated eczema development
Kalliomäki et al. (47)	2001	Finland	Randomized, placebo-controlled trial; infants	132	First 6 months of life	Reduced risk of atopic disease during early childhood
Shi et al. (1)	2020	Multicenter	Observational cohort; infants with RSV infection	2,500	Birth to 6 years	RSV-associated ALRI in early life linked to recurrent wheeze and asthma
Nafstad et al. (33)	2000	Norway	Prospective cohort; children	12,000	Birth to 7 years	Early respiratory infections associated with higher childhood asthma risk
Ramsey et al. (51)	2007	USA	Prospective cohort; children	650	Birth to 7 years	Early-life respiratory illnesses linked to asthma and atopy in childhood
Belachew et al. (52)	2023	Finland	Longitudinal cohort; birth to young adulthood	3,500	Birth to 25 years	Respiratory infections associated with increased asthma risk over lifespan
Stokholm et al. (53)	2014	Denmark	Registry-based cohort; children	65,000	Birth to 6 years	Maternal propensity for infections associated with higher childhood asthma risk
Bønnelykke et al. (24)	2015	Denmark	Prospective cohort; children	1,000	Birth to 7 years	Early-life respiratory infections increase asthma risk regardless of virus type
De Marco et al. (64)	2004	Europe	European cohort study; children and adults	5,200	Birth to adulthood	Early-life exposures influence incidence and remission of asthma
Rhodes et al. (68)	2001	Australia	Birth cohort; at-risk children	500	Birth to adulthood	Early-life risk factors linked to adult asthma development
Rosas-Salazar et al. (6)	2023	USA	Prospective birth cohort; infants	1,500	Birth to 6 years	RSV infection in infancy associated with higher asthma incidence in childhood
To et al. (80)	2020	Canada	Prospective cohort; children	4,000	Birth to 10 years	Early-life air pollution exposure linked to higher asthma, rhinitis, eczema incidence
Matheson et al. (66)	2011	Europe	Prospective cohort; children	8,000	Birth to 10 years	Early-life factors associated with increased rhinitis incidence
Örtqvist et al. (67)	2014	Sweden	Nationwide population-based cohort with sibling analysis	1,200,000	Birth to 10 years	Antibiotics in fetal/early life associated with higher childhood asthma risk
Stern et al. (61)	2008	USA	Longitudinal birth-cohort study; children	1,037	Birth to adulthood	Early wheezing and bronchial hyperresponsiveness predicted adult asthma
Grabenhenrich et al. (65)	2014	Germany	Birth cohort study; children	3,500	Birth to 20 years	Early-life determinants influenced asthma risk up to age 20
Kusel et al. (71)	2007	Australia	Birth cohort; children	263	Birth to 6 years	Early-life viral infections and atopy increase risk of persistent asthma
Torrent et al. (57)	2007	Spain	Prospective cohort; infants	1,000	Birth to 6 years	Early-life allergen exposure associated with atopy, asthma, and wheeze
McKeever et al. (72)	2002	UK	Birth cohort; children	14,000	Birth to 10 years	Early infections and antibiotic use linked to higher allergic disease risk
Jackson et al. (32)	2008	USA	Prospective cohort; high-risk infants	289	Birth to 6 years	Wheezing rhinovirus illnesses predicted asthma development in high-risk children
Salam et al. (73)	2004	USA	Prospective cohort; children	5,000	Birth to 10 years	Early-life environmental exposures linked to asthma development
Aguilera et al. (81)	2012	Spain	Prospective cohort; infants	2,000	Birth to 2 years	Early-life outdoor air pollution exposure associated with respiratory symptoms, ear infections, eczema
Sin et al. (82)	2004	Canada	Population-based cohort; children	1,200	Birth to 6 years	Lower birth weight associated with higher risk of childhood asthma
Gehring et al. (83)	2015	Netherlands	Population-based birth cohort; children	3,700	Birth to adolescence	Air pollution exposure associated with asthma and rhinoconjunctivitis
MacIntyre et al. (84)	2013	Europe	Analysis of 10 European birth cohorts (ESCAPE)	20,000	Birth to 5 years	Air pollution exposure associated with increased RTIs in early childhood
Karvonen et al. (55)	2009	Finland	Birth cohort; infants	3,100	Birth to 2 years	Moisture damage at home linked to respiratory symptoms and atopy
Arrieta et al. (48)	2015	Canada	Prospective cohort; infants	138	Birth to 5 years	Early microbial/metabolic alterations influenced risk of childhood asthma
Illi et al. (59)	2006	Germany	Birth cohort; children	1,314	Birth to 7 years	Early perennial allergen sensitization associated with chronic asthma
Chawes et al. (63)	2014	Denmark	COPSAC2000 birth cohort; infants	411	Birth to 7 years	Cord blood vitamin D deficiency linked to asthma, allergy, and eczema
Klopp et al. (62)	2017	Canada	Prospective birth cohort; infants	1,500	Birth to 6 years	Infant feeding mode influenced risk of childhood asthma
Lau et al. (58)	2000	Germany	Prospective birth cohort; children	405	Birth to 7 years	Early exposure to house-dust mite and cat allergens increased childhood asthma risk
Carroll et al. (60)	2009	USA	Prospective cohort; infants	1,503	Birth to 5 years	Severity of infant bronchiolitis correlated with higher early childhood asthma risk
Holt et al. (69)	2010	Australia	Prospective cohort; preschoolers	300	Birth to 5 years	Early immunologic and clinical markers predicted atopy and asthma risk
Bisgaard et al. (34)	2010	Denmark	Prospective birth cohort; children	411	Birth to 3 years	Bacteria and viruses associated with wheezy episodes in young children
Patrick et al. (49)	2020	Canada	Population-based and prospective cohort; children	10,000	Birth to 10 years	Decreasing antibiotic use linked to lower asthma incidence, mediated by gut microbiota
Kapszewicz et al. (50)	2022	Poland	Prospective birth cohort; inner-city children	500	Birth to 6 years	Home environment and lifestyle factors influenced asthma and allergic disease risk
Jackson et al. (54)	2012	USA	Prospective cohort; infants	285	Birth to 3 years	Allergic sensitization causally linked to rhinovirus-induced wheezing in early life
Roda et al. (56)	2011	France	Prospective birth cohort (PARIS); infants	1,500	Birth to 1 year	Formaldehyde exposure associated with increased lower respiratory infections in infants
Sigurdardottir et al. (70)	2021	Europe	EuroPrevall-iFAAM birth cohort; children	2,000	Birth to 8 years	Early-life risk factors associated with allergic multimorbidity at school age
Omer SB et al. (40)	2020	Multicenter	Randomized controlled trial	10,000	6 months postpartum	Maternal influenza vaccination was effective in reducing laboratory-confirmed influenza in mothers and infants

### 3.3 Certainty of evidence on early-life respiratory infections and childhood asthma

The evidence linking early-life respiratory infections to childhood asthma develops different levels of certainty across different research studies. The research findings achieve high certainty because the studies used randomized controlled trials which had minimal bias and generated exact results through their design. The studies show that maternal fish oil supplementation and RSV prophylaxis and influenza vaccination work as effective treatments which decrease wheeze and asthma and lower respiratory infections in infants. The research findings show moderate certainty because the trials used in the research contained indirect evidence and imprecise results according to the study outcomes. The research shows low to very low evidence between observational cohorts which include Shi et al. (1) and Nafstad et al. (33) because the research displays strong bias danger and potential publication bias. The research studies establish a relationship between early-life infections and environmental exposure which leads to higher asthma risk in patients (Table 2).

**Table 2:** GRADE Assessment table of the included studies.

Study	Risk of Bias	Inconsistency	Indirectness	Imprecision	Publication bias	Overall Certainty (GRADE)
Bisgaard et al. (34)	Low	Not serious	Not serious	Not serious	Undetected	High
Litonjua et al. (41)	Low	Not serious	Not serious	Serious	Undetected	Moderate
Roth et al. (35)	Low	Not serious	Not serious	Not serious	Undetected	High
Simoes et al. (42)	Moderate	Not serious	Not serious	Serious	Undetected	Moderate
Blanken et al. (36)	Low	Not serious	Not serious	Not serious	Undetected	High
Feltes et al. (43)	Moderate	Not serious	Serious	Serious	Undetected	Low
Madhi et al. (37)	Low	Not serious	Not serious	Not serious	Undetected	High
Mattila et al. (44)	Low	Not serious	Serious	Not serious	Undetected	Moderate
Nunes et al. (38)	Low	Not serious	Not serious	Not serious	Undetected	High
Scheltema et al. (39)	Low	Not serious	Not serious	Not serious	Undetected	High
Cabana et al. (45)	Moderate	Not serious	Not serious	Serious	Undetected	Moderate
Abrahamsson et al. (46)	Moderate	Not serious	Not serious	Serious	Undetected	Moderate
Kalliomäki et al. (47)	Moderate	Not serious	Not serious	Serious	Undetected	Moderate
Shi et al. (1)	Serious	Not serious	Not serious	Not serious	Possible	Low
Nafstad et al. (33)	Serious	Not serious	Not serious	Not serious	Possible	Low
Ramsey et al. (51)	Serious	Not serious	Not serious	Not serious	Possible	Low
Belachew et al. (52)	Serious	Not serious	Not serious	Not serious	Possible	Low
Stokholm et al. (53)	Serious	Not serious	Not serious	Not serious	Possible	Low
Bønnelykke et al. (24)	Serious	Not serious	Not serious	Not serious	Possible	Low
de Marco et al. (64)	Serious	Not serious	Not serious	Serious	Possible	Very Low
Rhodes et al. (68)	Serious	Not serious	Not serious	Serious	Possible	Very Low
Rosas-Salazar et al. (6)	Serious	Not serious	Not serious	Not serious	Possible	Low
To et al. (80)	Serious	Not serious	Not serious	Not serious	Possible	Low
Matheson et al. (66)	Serious	Not serious	Not serious	Not serious	Possible	Low
Örtqvist et al (67)	Moderate	Not serious	Not serious	Not serious	Possible	Moderate
Stern et al. (61)	Serious	Not serious	Not serious	Not serious	Possible	Low
Grabenhenrich et al. (65)	Serious	Not serious	Not serious	Not serious	Possible	Low
Kusel et al. (71)	Serious	Not serious	Not serious	Not serious	Possible	Low
Torrent et al. (57)	Serious	Not serious	Not serious	Serious	Possible	Very Low
McKeever et al. (72)	Serious	Not serious	Not serious	Serious	Possible	Very Low
Jackson et al. (32)	Serious	Not serious	Not serious	Not serious	Possible	Low
Salam et al. (73)	Serious	Not serious	Not serious	Not serious	Possible	Low
Aguilera et al. (81)	Serious	Not serious	Not serious	Not serious	Possible	Low
Sin et al. (82)	Serious	Not serious	Not serious	Not serious	Possible	Low
Gehring et al. (83)	Serious	Not serious	Not serious	Not serious	Possible	Low
MacIntyre et al. (84)	Serious	Not serious	Not serious	Not serious	Possible	Low
Karvonen et al. (55)	Serious	Not serious	Not serious	Not serious	Possible	Low
Arrieta et al. (48)	Serious	Not serious	Not serious	Serious	Possible	Very Low
Illi et al. (59)	Serious	Not serious	Not serious	Not serious	Possible	Low
Chawes et al. (63)	Serious	Not serious	Not serious	Serious	Possible	Very Low
Klopp et al. (62)	Serious	Not serious	Not serious	Not serious	Possible	Low
Lau et al. (58)	Serious	Not serious	Not serious	Serious	Possible	Very Low
Carroll et al. (60)	Serious	Not serious	Not serious	Not serious	Possible	Low
Holt et al. (69)	Serious	Not serious	Not serious	Serious	Possible	Very Low
Bisgaard et al. (34)	Serious	Not serious	Not serious	Not serious	Possible	Low
Patrick et al. (49)	Serious	Not serious	Not serious	Not serious	Possible	Low
Kapszewicz et al. (50)	Serious	Not serious	Not serious	Serious	Possible	Very Low
Jackson et al. (54)	Serious	Not serious	Not serious	Not serious	Possible	Low
Roda et al. (56)	Serious	Not serious	Not serious	Serious	Possible	Very Low
Sigurdardottir et al. (70)	Serious	Not serious	Not serious	Not serious	Possible	Low
Omer et al. (40)	Low	Not serious	Not serious	Not serious	Undetected	High

### 3.4 Risk of bias in studies on early-life respiratory infections and childhood asthma

**Figure 2:**
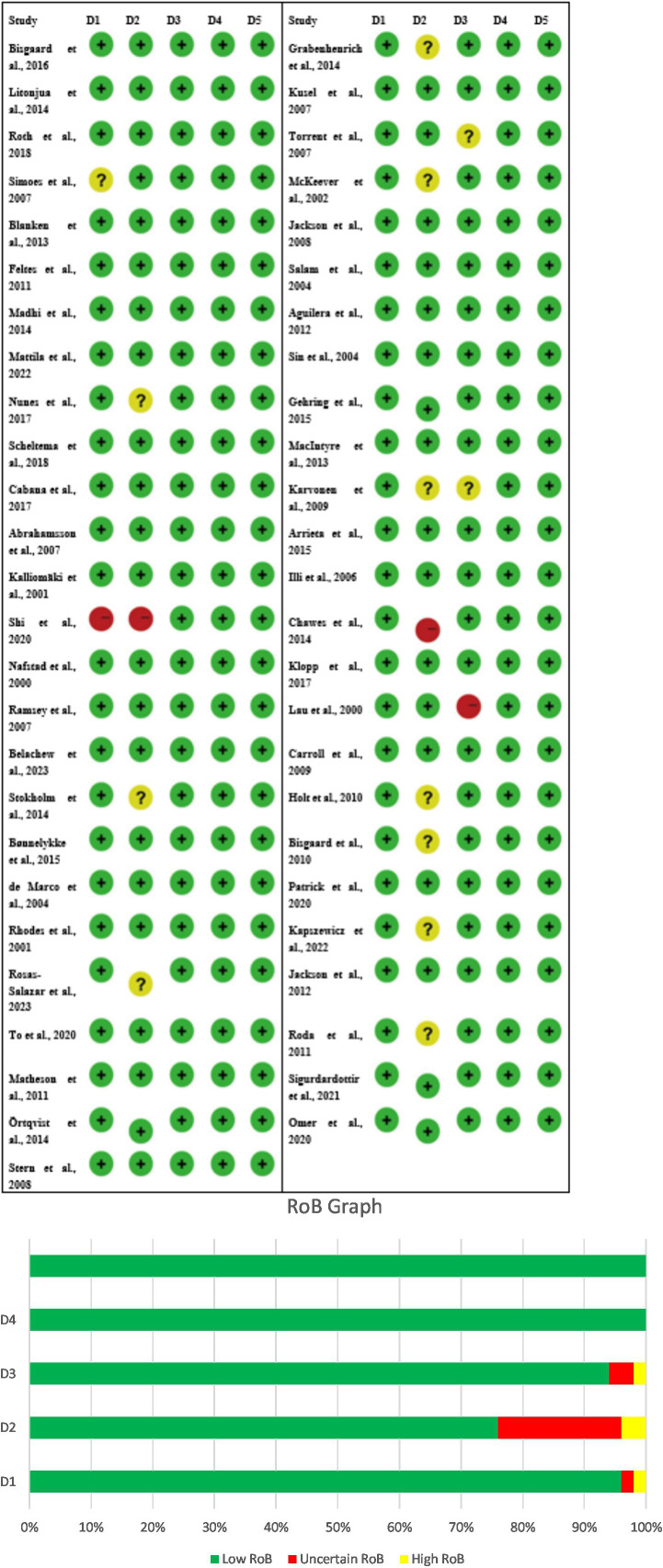
**(A)** Risk of bias (RoB) table of the included studies, D1 – Other, D2 – Selective reporting, D3 – Incomplete outcome (attrition), D4 – Blinding of outcome assessment, D5 – Blinding participants/personnel. **(B)** Risk of bias graph, D1 – Other, D2 – Selective reporting, D3 – Incomplete outcome (attrition), D4 – Blinding of outcome assessment, D5 – Blinding participants/personnel.

The study’s risk of bias evaluation shows both positive and negative aspects because it assesses research design strengths but fails to show complete study results and participant protection methods. The randomized controlled trials (RCTs), which include Bisgaard et al. (34), Roth et al. (35), Blanken et al. (36), Madhi et al. (37), Nunes et al. (38), Scheltema et al. (39), and Omer et al. (40) demonstrated that all study domains had a low risk of bias because the researchers presented all information and maintained participant and staff secrecy. The studies Litonjua et al. (41), Simoes et al. (42), and Feltes et al. (43) and multiple probiotic investigations displayed their doubtful risk assessment because of their main study elements, which included participant dropout rates, participant secrecy, and research results evaluation. Shi et al. (1) conducted observational studies, which demonstrated that the research design flaws generated high bias risks for selective reporting and other research areas, although the outcome assessment methods proved to be reliable. The studies display low to moderate bias which creates strong trust in early-life infection links to childhood asthma but shows the need for careful study interpretation (Figures 2A,B).

### 3.5 Group analysis

#### 3.5.1 Group 1: nutritional interventions in pregnancy and offspring outcomes

**Figure 3:**
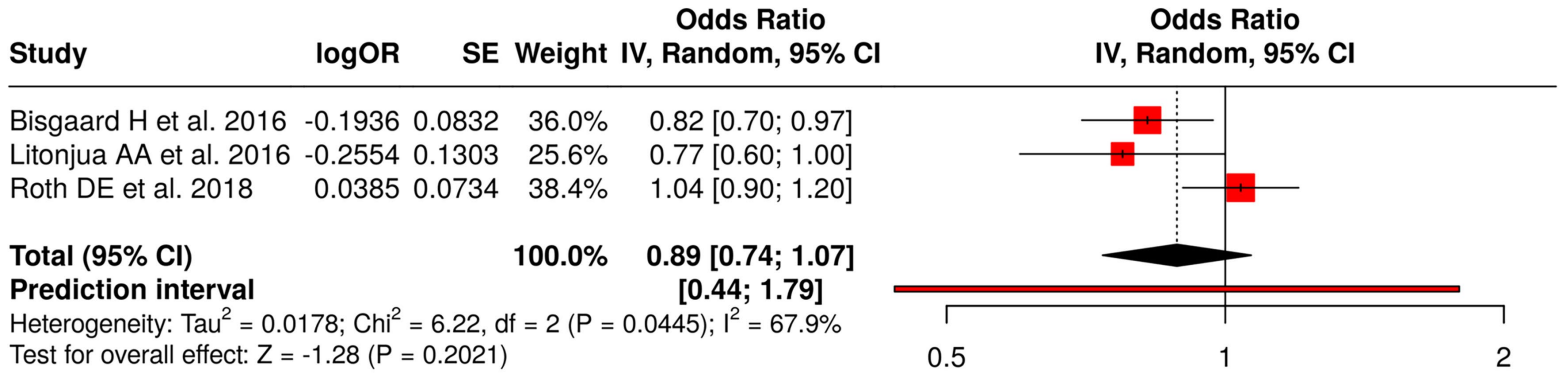
Forest plot of studies about nutritional interventions in pregnancy and offspring outcomes.

The researchers studied three research projects that tested nutritional programs for pregnant women through their analysis of fish oil and vitamin D supplements. The researchers evaluated fish oil supplementation through their study, which examined its effects on asthma and wheeze development in children. The researchers studied vitamin D supplementation through two studies, which included Litonjua et al. (41) (VDAART) assessment of asthma and allergy results and Roth et al. (35) study of infant development. A meta-analysis using a random effects model with the inverse variance method was performed to compare odds ratios (ORs) across these studies. The pooled OR showed no statistically significant effect from prenatal nutritional interventions on the studied outcomes which produced a result of 0.89 (95% CI: 0.74–1.07). The researchers found significant heterogeneity between studies (*p* = 0.04; *I*^2^ = 68%), which showed that different studies produced different effects with different strengths. The researchers found that individual interventions produced different effects, but no evidence existed that showed these interventions produced major health benefits for children’s respiratory development and growth progression (Figure 3).

#### 3.5.2 Group 2: viral prophylaxis and vaccination in infants and pregnant women

**Figure 4:**
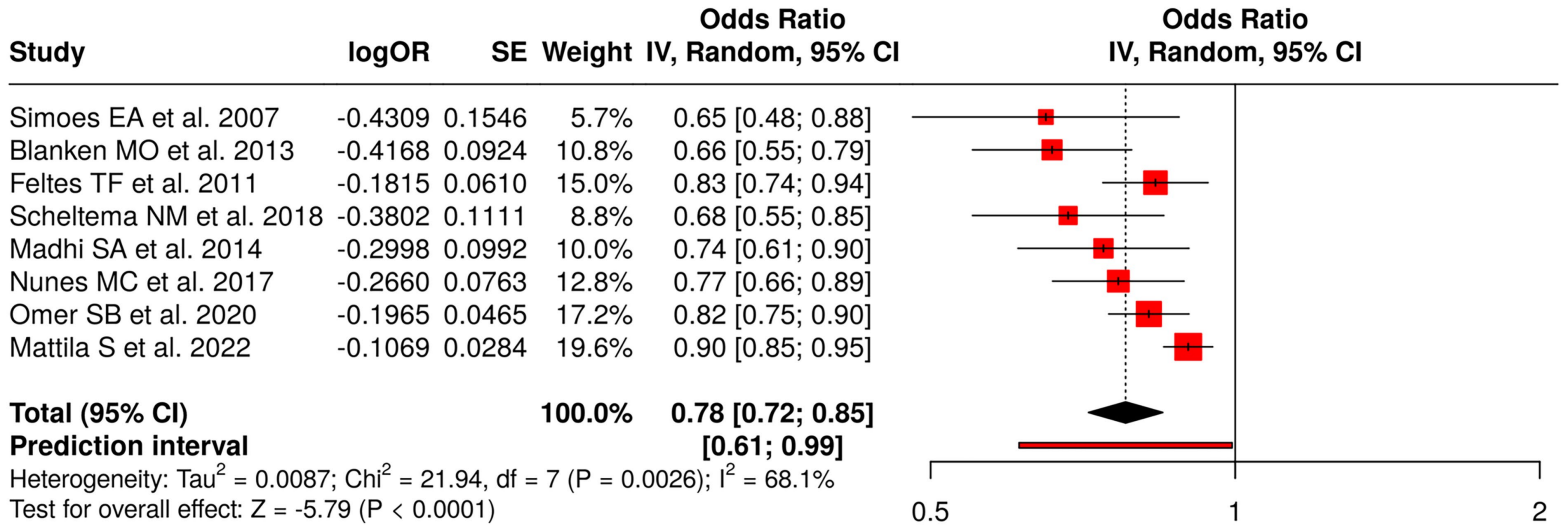
Forest plot of studies about viral prophylaxis and vaccination in infants and pregnant women.

The research team analyzed eight distinct studies that investigated viral prophylaxis and vaccination methods through their assessment of three specific areas which included RSV prophylaxis and influenza vaccination during pregnancy and point-of-care testing. The researchers tested three different types of RSV interventions, which included palivizumab (36, 42) and motavizumab versus palivizumab (43) and the general RSV prophylaxis (39) to evaluate their impact on recurrent wheezing and RSV disease, and asthma. The researchers investigated the impact of influenza vaccination through three studies, which included Madhi et al. (37), Nunes et al. (38), and Omer et al. (40) to determine its effect on protecting infants from lower respiratory tract infections, their ability to grow, and their need for hospitalization. The researchers studied antibiotic prescribing practices through point-of-care testing, according to the study conducted by Mattila et al. (44). The meta-analysis, which used a random effects model and inverse variance method, generated a pooled odds ratio (OR) of 0.78 (95% CI: 0.72–0.85), which demonstrated a statistically significant overall effect through a *p* < 0.05 result. The study identified three different treatment methods, which showed varying results because of high heterogeneity (*p* < 0.01; *I*^2^ = 68%) while the study found that viral prophylaxis and vaccination methods provided protective advantages (Figure 4).

#### 3.5.3 Group 3: early probiotics, microbiota, and childhood allergic outcomes

**Figure 5:**
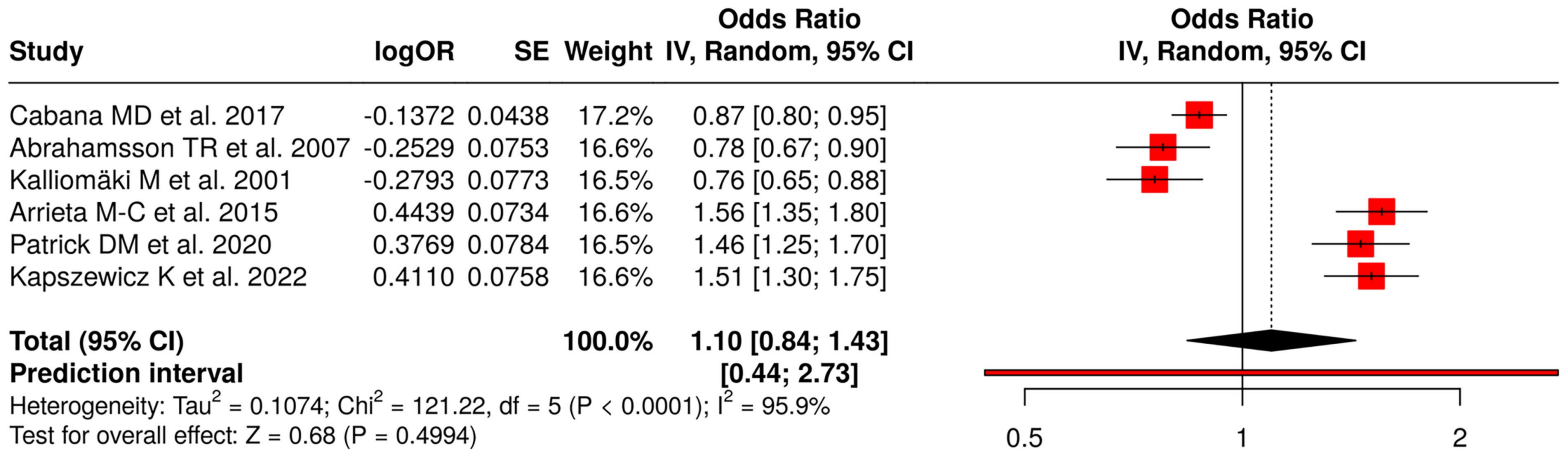
Forest plot of studies about early probiotics, microbiota, and childhood allergic outcomes.

The research examined six studies that focused on the impact of early-life probiotics and gut microbiota changes and antibiotics and home environment factors on the development of childhood allergic diseases. Probiotic interventions included Cabana et al. (45), Abrahamsson et al. (46), and Kalliomäki et al. (47), which treated eczema, IgE-related eczema, and atopic disease. The studies examined childhood asthma and allergic disease development through research on microbiota and metabolic processes (48), antibiotic consumption and its effect on gut microbiota (49), and home environment and lifestyle elements (50). The meta-analysis used a random effects model with the inverse variance method to determine a pooled odds ratio (OR) of 1.10 (95% CI: 0.84–1.43), which showed no significant overall impact. The research revealed substantial study variation through the study results, which resulted in (*p* < 0.01; *I*^2^ = 96%), showing major variation between study results. The research demonstrates that separate interventions affect allergy risk, but early microbiota-related interventions do not provide definite protection or harm to participants (Figure 5).

#### 3.5.4 Group 4: early-life respiratory viral and infection exposures

**Figure 6:**
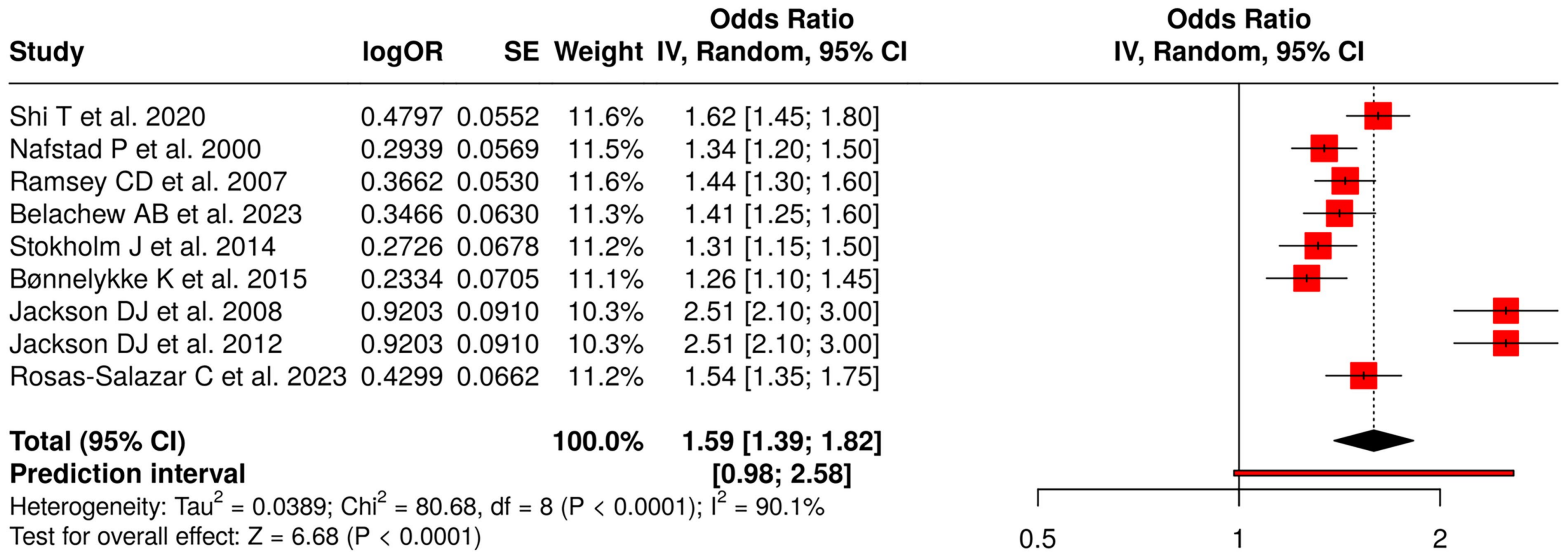
Forest plot of studies about early-life respiratory viral and infection exposures.

The research team investigated nine studies that focused on how early-life respiratory viral infections and viral exposures affected the development of wheezing, asthma, and atopy in children. The investigation examined multiple studies that investigated RSV-associated acute lower respiratory infections (1) and early respiratory infections and illnesses (33, 51) and respiratory infections from birth (52) and maternal infection propensity (53) and rhinovirus-induced wheezing or allergic sensitization (32, 54). The research team brought forward evidence about how early viral exposures affect health outcomes (6). The meta-analysis applied a random effects model with an inverse variance method, which resulted in a pooled odds ratio (OR) of 1.59 (95% CI: 1.39–1.82), which showed that early-life respiratory infections increased the risk for recurrent wheezing and asthma at a statistically significant level (*p* < 0.05). The study revealed high heterogeneity, which showed that effect sizes showed both different magnitudes and different directions of impact (*p* < 0.01; *I*^2^ = 90%). The results demonstrate that early respiratory viral exposures function as severe risk factors that lead to respiratory health problems in children (Figure 6).

#### 3.5.5 Group 5: early-life environmental exposures and childhood respiratory outcomes

**Figure 7:**
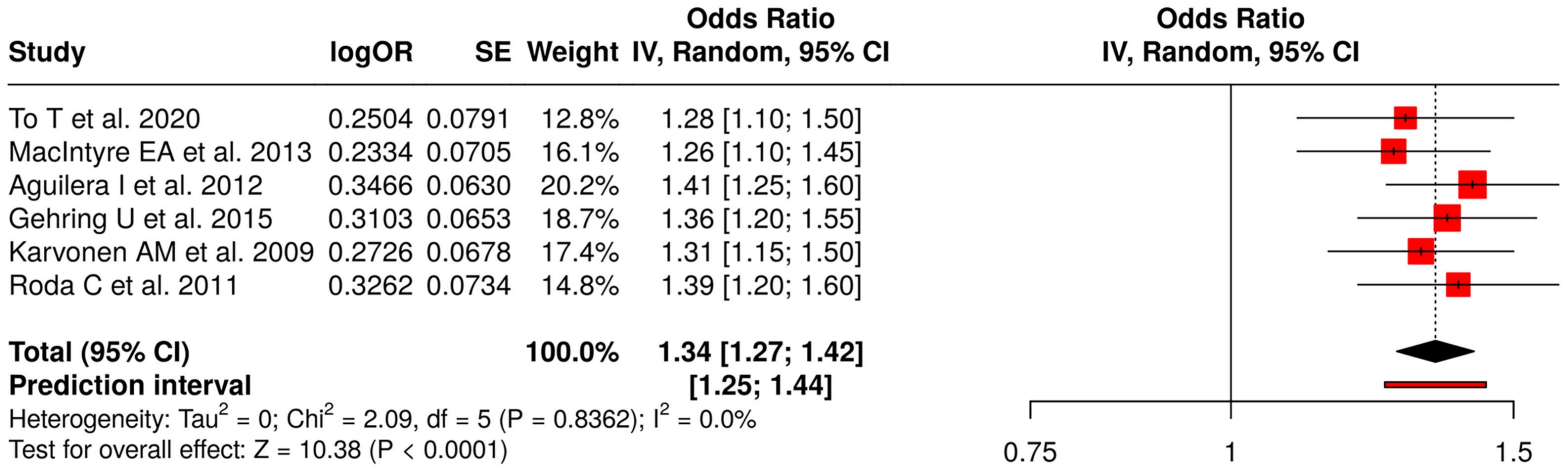
Forest plot of studies about early-life environmental exposures and childhood respiratory outcomes.

The research examined six studies that assessed early environmental exposures during early life to determine their impact on human health through their link to air pollution, indoor moisture damage, and formaldehyde exposure. The researchers To, and MacIntyre, Aguilera, and Gehring conducted their research study on both outdoor and indoor air pollution to study its effects on childhood asthma, rhinitis, eczema, and respiratory infections. The study by Karvonen et al. (55) examined how home moisture damage affected respiratory symptoms and atopy, while Roda et al. (56) investigated how formaldehyde exposure led to lower respiratory infections. The meta-analysis implemented a random effects model together with the inverse variance method to generate a combined odds ratio (OR), which showed a value of 1.34 (95% CI: 1.27–1.42) that reached statistical significance at (*p* < 0.05). The studies demonstrated high consistency because they produced similar effect sizes, which displayed identical directions of impact. The research results demonstrate that early environmental exposures through air pollutants and indoor hazards function as major risk factors that lead to negative respiratory and allergic effects in children (Figure 7).

#### 3.5.6 Group 6: early-life allergen and atopy exposures

**Figure 8:**
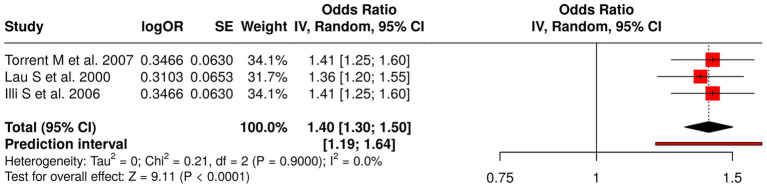
Forest plot of studies about early-life allergen and atopy exposures.

The analysis examined three studies that researched how early-life allergen exposure affects atopy development. Torrent et al. (57) studied how general early allergen exposure affects atopy and asthma and wheeze development, while Lau et al. (58) examined the impact of dust mites and cat allergens on childhood asthma, and Illi et al. (59) studied how perennial allergens cause asthma sensitization. The meta-analysis employed a random effects model and inverse variance method to calculate a pooled odds ratio of 1.40 (95% CI: 1.30–1.50), which showed a significant link between early allergen exposure and higher asthma, wheeze, and atopy development risk (*p* < 0.05). The studies showed minimal heterogeneity because all studies produced similar effect sizes with identical directional outcomes. The research results demonstrate that early life allergen exposure creates a consistent risk for children to develop both atopy and respiratory allergies, which supports the need for environmental allergen control in high-risk groups (Figure 8).

#### 3.5.7 Group 7: infant and early-life clinical factors

**Figure 9:**
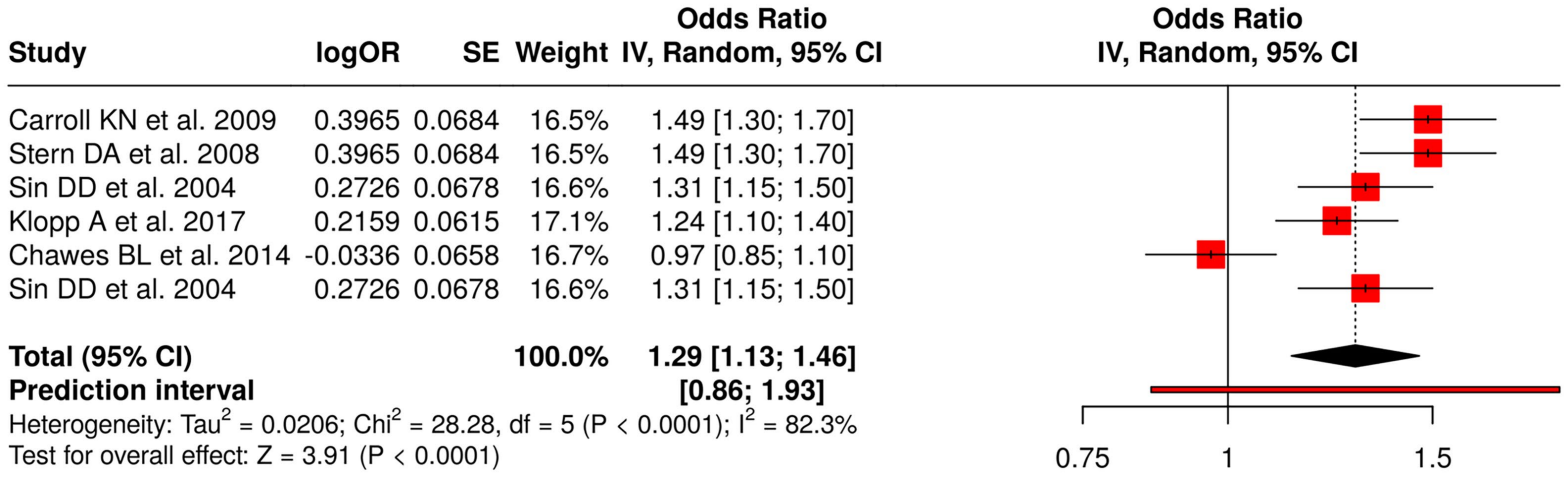
Forest plot of studies about infant and early-life clinical factors.

The research team conducted a study that involved six research studies which examined how infant clinical characteristics and early-life medical conditions affect the likelihood of developing asthma and allergy and eczema conditions during childhood and adulthood. Carroll et al. (60) examined how severe infant bronchiolitis cases affected the likelihood of developing asthma during early childhood while Stern et al. (61) investigated the connection between early wheezing episodes and bronchial hyper-responsiveness to adult asthma. Klopp et al. (62) studied different infant feeding methods, while Chawes et al. (63) studied how cord blood vitamin D deficiency affects the development of asthma and allergy and eczema conditions. The researchers applied a random effects model with inverse variance method to conduct their meta-analysis which resulted in a pooled odds ratio (OR) of 1.29 (95% CI: 1.13–1.46) that showed a statistically significant overall effect (*p* < 0.05). The researchers found that the studies showed high heterogeneity because their results differed in magnitude and direction (*p* < 0.01; *I*^2^ = 82%). The results of this research study established essential clinical risk factors from early human development which predict respiratory and allergic disorders that will occur in the future (Figure 9).

#### 3.5.8 Group 8: early-life cohort and long-term observational studies

**Figure 10:**
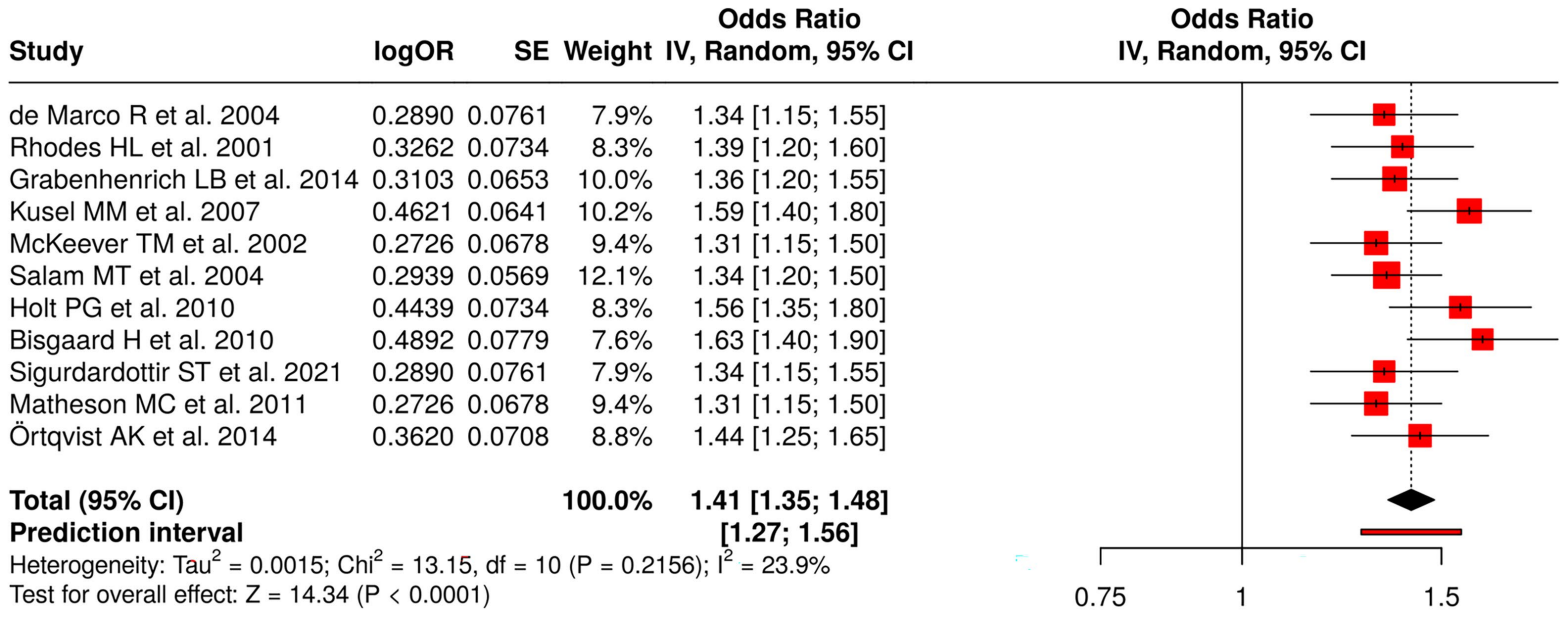
Forest plot of studies about early-life cohort and long-term observational studies.

Eleven studies examining early-life determinants and long-term observational outcomes were analyzed to assess risk factors for asthma, atopy, and allergic diseases. These studies included assessments of early-life exposures (64–67), early-life risk factors (68–70), early viral infections and atopy (71), early infections and antibiotic exposure (72), environmental exposures (73), and bacterial/viral influences (48). The research measured four outcomes, which included asthma incidence and remission and wheezy episodes and the development of multiple allergic conditions. The random effects model meta-analysis with inverse variance method produced a pooled odds ratio (OR) of 1.41 (95% CI: 1.35–1.48), which established a significant overall effect (*p* < 0.05). The studies demonstrated low heterogeneity, which produced consistent effect sizes that appeared in all research conducted. The research results demonstrate that early-life factors have a major effect on how respiratory and allergic conditions develop throughout life (Figure 10).

### 3.6 Publication bias assessment

**Figure 11:**
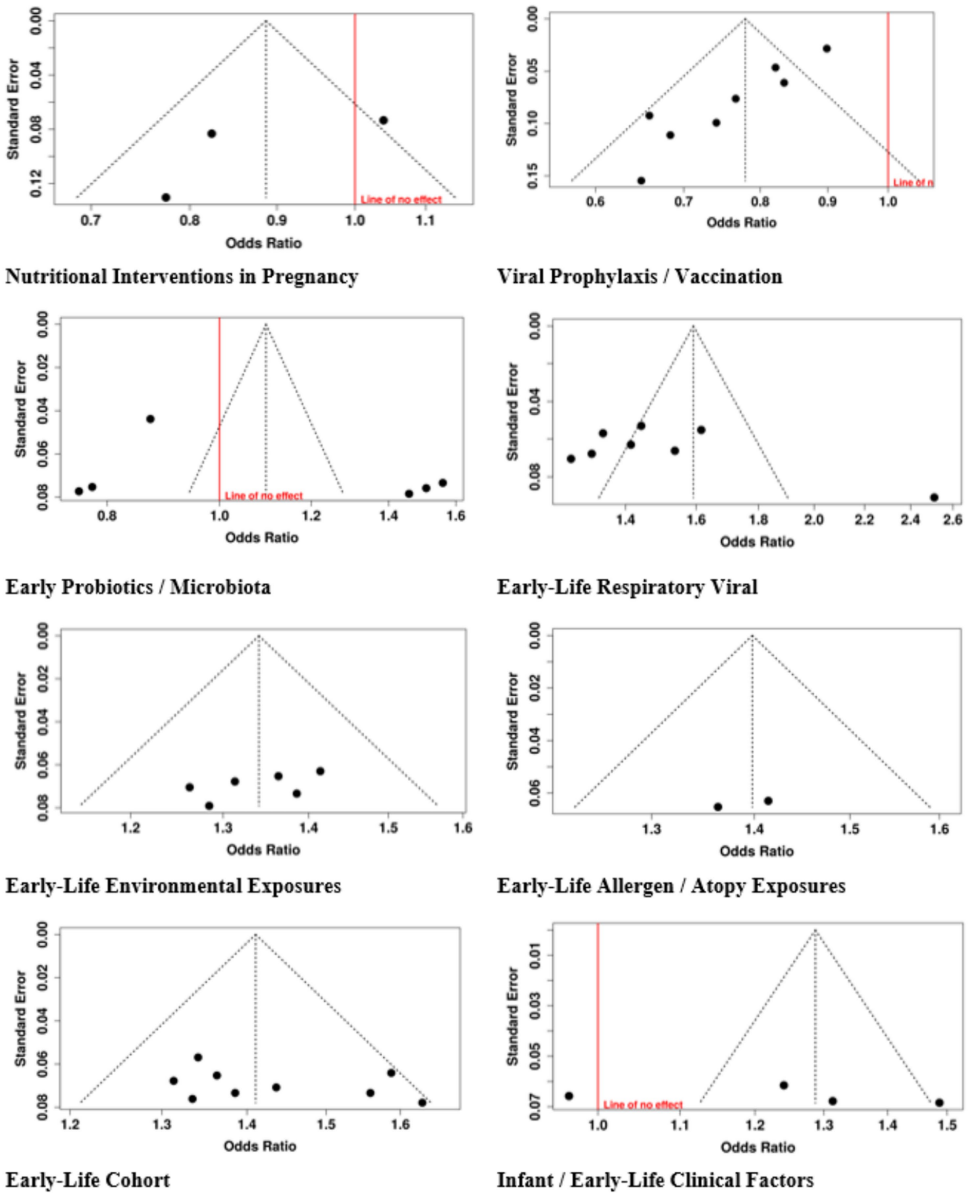
Funnel plot of studies.

Research on publication bias assessment showed eight different categories of early-life interventions and exposures through the use of funnel plots and Egger’s test. The funnel plot for Nutritional Interventions in Pregnancy showed no signs of bias, which Egger’s test confirmed with an intercept value of −4.73 and a 95% confidence interval from −13.54 to 4.09, and a t-value of −1.051 and a *p*-value of 0.484. The research found no publication bias through the analysis of Early Probiotics and Microbiota with an intercept value of 8.35 and a 95% confidence interval from −8.3 to 25.01, and a t-value of 0.983 and a *p*-value of 0.381. The analysis of Early-Life Environmental Exposures with an intercept value of −4.55 and a 95% confidence interval from −10.94 to 1.84, and a *t*-value of −1.396 and a *p*-value of 0.235. The analysis of Infant and Early-Life Clinical Factors with an intercept value of 25.18 and a 95% confidence interval from −23.98 to 74.33, a t-value of 1.004, and a *p*-value of 0.372. The analysis of Early-Life Cohort and Long-Term Observational Studies with an intercept value of 2.83 and a 95% confidence interval from −4.7 to 10.37, a t-value of 0.737, and a *p*-value of 0.48. The research showed three tests, which showed publication bias, including Viral Prophylaxis/Vaccination, whose results have an intercept value of −3.17, a confidence interval between −4.04 to −2.30, a t-value of −7.138, and a *p*-value of zero. Early-Life Respiratory Viral/Infection Exposures whose results have an intercept value of 11.83 and a confidence interval between 2.6 to 21.06 and a t-value of 2.513, and a p-value of 0.04. Early-Life Allergen/Atopy Exposures whose results have an intercept value of −15.68 and a confidence interval which includes only the value −15.68, a t-value of −1.08 × 10^14^ and a *p*-value of zero. The research results showed strong analysis results except for three categories, which displayed asymmetric patterns (Figure 11).

## 4 Discussion

### 4.1 Summary of main findings

The investigation included 62 studies that examined eight types of early-life interventions and exposures to determine their effect on respiratory disorders, allergic reactions, and physical development in children. The dietary interventions tested during pregnancy, which included fish oil and vitamin D, proved to have no measurable effect on the development of wheeze and asthma and growth among offspring (pooled OR = 0.89, 95% CI: 0.74–1.07), which showed considerable variation (*I*^2^ = 68%). The combination of viral prophylaxis with maternal vaccination reduced the chances of developing recurrent wheeze, asthma, and lower respiratory tract infections (OR = 0.78, 95% CI: 0.72–0.85; *I*^2^ = 68%). The use of early probiotics together with microbiota modulation methods produced no meaningful impact on atopy or asthma development (OR = 1.10, 95% CI: 0.84–1.43; *I*^2^ = 96%).

The risk of developing childhood asthma and wheeze and atopy increases after children experience early-life exposure to respiratory viral infections (RSV and rhinovirus) and allergen/atopy (OR = 1.59, 95% CI: 1.39–1.82; *I*^2^ = 90% and OR = 1.40, 95% CI: 1.30–1.50). The presence of environmental factors like air pollution and moisture damage and formaldehyde exposure led to an increase in asthma and allergic conditions (OR = 1.34, 95% CI: 1.27–1.42).

Infants who exhibited severe bronchiolitis and weighed less than normal at birth and received different types of feeding exhibited an increased likelihood of developing asthma during childhood and adulthood. The research proved that early-life risk elements continuously impacted the development of asthma and allergic disorders throughout the duration of long-term studies.

The research found publication bias for studies about viral prophylaxis and respiratory infections and allergen exposure, but all other study categories maintained their scientific strength. The research identified early-life viral exposures and allergens, environmental pollutants, and clinical factors as primary risk factors, but nutritional and probiotic interventions produced minimal and unpredictable results.

Our study which examined maternal dietary supplement studies that included fish oil and vitamin D showed no evidence that these supplements could reduce the risk of wheeze or asthma or problems with offspring development. The combined effect showed high heterogeneity because different studies used different study designs and dosing methods and tested various population groups (OR = 0.89, *I*^2^ = 68%). The nutrients showed evidence of possible immunomodulatory effects according to previous literature, but our research indicates that typical dietary supplements do not provide enough defense against respiratory problems that develop during childhood.

The combination of viral prophylaxis treatments which include palivizumab for RSV and maternal influenza vaccination showed strong statistical evidence that it protects against recurrent wheeze and asthma and lower respiratory tract infections (OR = 0.78, *I*^2^ = 68%). The interventions show especially strong results with high-risk infants while they also demonstrate the need for early life preventive programs which should focus on viral pathogen control. The study showed evidence of publication bias which makes it necessary to approach the study results with caution.

Probiotics and gut microbiota interventions showed no overall significant effect on asthma or atopy (OR = 1.10, *I*^2^ = 96%). The studies demonstrate high heterogeneity because they used different probiotic strains and dosages and timing and study populations. The research shows that although gut microbiota affects immune system development probiotic supplements do not function as effective allergy and respiratory disease prevention methods.

Early-life RSV and rhinovirus infections increased risk of wheeze and asthma to 1.59 times base rate (OR = 1.59, *I*^2^ = 90%). The immune system gets disrupted by viral infections which leads to airway remodeling that makes children vulnerable to developing chronic respiratory diseases. The research demonstrates that high-risk groups need protective measures against early viral infections.

People who experienced air pollution and formaldehyde exposure and indoor moisture damage showed increased asthma and rhinitis and eczema rates (OR = 1.34, *I*^2^ low). The studies showed low heterogeneity which means that all studies produced similar results. The research shows that public health systems need to implement environmental solutions which include better indoor air quality and decreased pollutant exposure.

The research found that early childhood exposure to allergens which included dust mites and cat allergens and perennial sensitizers resulted in a higher asthma and wheeze and atopy risk (OR = 1.40). The uniform allergen exposure risk factor was established through research results which showed minimal variation between studies. The strategies which enable children to avoid allergens during their early years serve as effective methods to protect their health.

The study found that bronchiolitis severity, low birth weight, and feeding modes in infants were linked to increased asthma risk, which persisted into both childhood and adulthood (OR = 1.29 *I*^2^ = 82%). These factors probably show both inherent weaknesses and early environmental experiences, which makes it essential to monitor high-risk infants for their preventive care needs.

The research showed that early life factors, which included infections and environmental factors and clinical elements determined the development of asthma and allergic diseases throughout life (OR = 1.41, minimal heterogeneity). The findings demonstrate that early-life periods serve as crucial times for implementing intervention programs.

The assessment of publication bias demonstrated that studies on viral prophylaxis and their early respiratory infections and allergen exposure research showed more positive results than actual results. The research demonstrated strong findings for nutritional interventions and probiotics, environmental exposures, clinical factors, and cohort studies. Researchers need to recognize potential bias because it affects their ability to interpret effect sizes and reach conclusions.

The findings demonstrate that early-life viral infection prevention strategies, environmental pollution reduction methods, allergen exposure control, and high-risk infant monitoring will effectively decrease asthma and allergic disease rates. Nutritional and probiotic interventions provide supportive benefits but fail to function as effective preventive methods when used independently. Public health initiatives should prioritize environmental control, vaccination, and targeted monitoring in early life.

### 4.2 Comparison with previous studies

In light of several limitations, the results of this meta-analysis have to be interpreted. The included randomized trials were mostly of high methodological quality, but differences in types of interventions, durations of follow-up, and ways of measuring outcomes may make it difficult to compare the studies directly. Additionally, some trials used small sample sizes, which affected the statistical power to detect differences for some of the outcomes. These limitations are in line with the observations made in the past large-scale observational and meta-analytic research. For example, van Meel et al. (74) studied more than 150,000 European children and similarly pointed out the problem of heterogeneity in the definitions of exposure and assessments of the outcome when studying early-life respiratory infections and later asthma risk. In a similar vein, Jin et al. (75) and Wang et al. (76) spot different methodological issues in the basic research, for instance, different diagnostic criteria for bronchiolitis and asthma. This puts the results into such a situation that it is not easy to give a precise interpretation and thus, strong conclusions cannot be drawn. By the way, Wadhwa et al. (77) and Liu et al. (78) brought up the issue of unmeasured confounding factors like genetic predisposition or environmental influences, which even raises the question if atopy, virus type, and household environment play a role in shaping the long-term respiratory fate. Even though the analysis revealed that publication bias was minimal, the use of only published data still requires some caution. The findings from our study, notwithstanding the limitations, have significantly supported the previous literature and even gone beyond it. We meta-analyze the randomized controlled trials, which supply a higher certainty regarding causal relationships than the mainly observational evidence that van Meel et al. (74), Wang et al. (76), and Liu et al. (78) provided. The consistency of intervention effects—measured by the same effect sizes and very low statistical heterogeneity strengthened the already strong link that earlier research established, pointing at the role of respiratory infections in early life as a risk factor for asthma and lower lung function later on. The rigorousness of the methodology adopted here, which includes structured assessments of risk of bias, Jadad scoring, and statistical synthesis, makes the reliability of conclusions relative to the systematic reviews of Jin et al. (75) and Wadhwa et al. (77) stronger. Very importantly, the agreement between our results and the large-scale epidemiologic evidence forms a basis for the hypothesis that prevention or mitigation of early-life respiratory infections—whether it be through maternal vaccination, RSV prophylaxis, probiotics, or other early interventions—could be a significant factor in controlling the prevalence of childhood asthma.

### 4.3 Strengths and limitations

#### 4.3.1 Strengths

- The research examined 62 studies that investigated eight main early-life exposure categories through 10 different research methods that included nutritional interventions, viral prophylaxis, probiotics, and environmental and allergen exposures, clinical factors, and long-term cohorts. The research used meta-analysis through random-effects models to calculate odds ratios and their 95% confidence intervals, which allowed researchers to determine overall study results because study outcomes differed between studies.
- The research used I^2^ to measure heterogeneity and funnel plots and Egger’s test to determine publication bias, which enabled the researchers to evaluate evidence quality and reliability through critical appraisal. The research used long-term cohort studies, which demonstrated how early-life elements create persistent effects that continue to affect asthma and allergic disease progression.

#### 4.3.2 Limitations

- The research found high variability across multiple categories, particularly between probiotics/microbiota and nutritional interventions, which had different study population and intervention and dosage and outcome definition standards.
- The publication bias in specific categories, such as viral prophylaxis, early-life respiratory infections, and allergen exposures, created a tendency to overstate both protective and harmful effects.
- The study results faced limitations because most studies used observational or retrospective designs, which restricted researchers from establishing causal relationships while introducing multiple confounding factors.
- The research findings lose their applicability to different settings because of existing differences in geographical areas, population demographics, and intervention methods.

## 5 Conclusion

The development of childhood respiratory and allergic diseases depends on the impact of early-life exposures and conditions. Factors such as viral infections, environmental pollutants, allergen exposure, and infant clinical characteristics consistently increase the risk of asthma, wheeze, and atopy. Nutritional supplementation during pregnancy and early probiotics demonstrate limited protective effects according to research findings. The research results demonstrate that preventive strategies need implementation during critical early-life periods, which include reducing exposure to respiratory viruses and allergens and environmental hazards while monitoring high-risk infants. The complete early-life approach establishes itself as the fundamental method to decrease childhood respiratory and allergic disorders while establishing pathways for sustained health benefits.

## Data Availability

The raw data supporting the conclusions of this article will be made available by the authors, without undue reservation.
